# Using large language models for extracting and pre-annotating texts on mental health from noisy data in a low-resource language

**DOI:** 10.7717/peerj-cs.2395

**Published:** 2024-11-28

**Authors:** Sergei Koltcov, Anton Surkov, Olessia Koltsova, Vera Ignatenko

**Affiliations:** Laboratory for Social & Cognitive Informatics, National Research University Higher School of Economics, St. Petersburg, Russia

**Keywords:** Zero shot classification, Large language model, Natural Language Inference (NLI), Psychological text data

## Abstract

Recent advancements in large language models (LLMs) have opened new possibilities for developing conversational agents (CAs) in various subfields of mental healthcare. However, this progress is hindered by limited access to high-quality training data, often due to privacy concerns and high annotation costs for low-resource languages. A potential solution is to create human-AI annotation systems that utilize extensive public domain user-to-user and user-to-professional discussions on social media. These discussions, however, are extremely noisy, necessitating the adaptation of LLMs for fully automatic cleaning and pre-classification to reduce human annotation effort. To date, research on LLM-based annotation in the mental health domain is extremely scarce. In this article, we explore the potential of zero-shot classification using four LLMs to select and pre-classify texts into topics representing psychiatric disorders, in order to facilitate the future development of CAs for disorder-specific counseling. We use 64,404 Russian-language texts from online discussion threads labeled with seven most commonly discussed disorders: depression, neurosis, paranoia, anxiety disorder, bipolar disorder, obsessive-compulsive disorder, and borderline personality disorder. Our research shows that while preliminary data filtering using zero-shot technology slightly improves classification, LLM fine-tuning makes a far larger contribution to its quality. Both standard and natural language inference (NLI) modes of fine-tuning increase classification accuracy by more than three times compared to non-fine-tuned training with preliminarily filtered data. Although NLI fine-tuning achieves slightly higher accuracy (0.64) than the standard approach, it is six times slower, indicating a need for further experimentation with NLI hypothesis engineering. Additionally, we demonstrate that lemmatization does not affect classification quality and that multilingual models using texts in their original language perform slightly better than English-only models using automatically translated texts. Finally, we introduce our dataset and model as the first openly available Russian-language resource for developing conversational agents in the domain of mental health counseling.

## Introduction

Artificial intelligence, particularly large language models (LLMs), is increasingly being explored as a tool for various mental health care tasks within both psychiatry and psychology, as is shown in multiple recent reviews (Guo et al., 2024; Haque & Rubya, 2022; He et al., 2023; Hua et al., 2024; Li, 2024; Obradovich et al., 2024; Volkmer, Meyer-Lindenberg & Schwarz, 2024; Yang et al., 2023). These tasks can be broadly categorized into several areas: automatic detection of psychiatric disorders, psychological conditions, or personality traits from diverse data sources; generation of treatment recommendations for mental health practitioners; and support for mental health care seekers through automatic dialogue systems or conversational agents (CAs) designed to provide advice, counseling, and psychotherapeutic sessions.

The success of AI systems in addressing these tasks, however, heavily depends on the availability and quality of training data, which poses a significant challenge, particularly for low-resource languages and societies. While diagnostic systems can, in principle, utilize any data types as long as the correct diagnoses are present in the training sets, dialogue systems require question-answer text pairs whose relevance and quality have been validated by experts. In cases where conversations between mental health seekers and providers are unavailable, one approach is to label texts from open sources, such as online user-user or user-professional forums. However, the proportion of high-quality or even relevant texts in these data sets is so low that manual assessment would be highly inefficient, if not impossible. This situation creates a demand for automatic pre-filtering to eliminate irrelevant texts and for pre-annotation or pre-classification of the remaining texts into meaningful categories, such as discussions of specific disorders and psychological conditions. Once enriched in this manner, text collections can be more efficiently hand-labeled in terms of their topics, the quality of responses, and other important features. Social media data provide an opportunity to train models for pre-annotation tasks since they often contain user labels that, albeit loosely, are able to indicate certain text classes, such as topic, sentiment or communication purpose.

In this work, we address the task of pre-filtering and pre-annotating of noisy online data for the subsequent training of mental health care conversational agents by exploring the capabilities of LLMs operating in zero-shot classification mode. One potential application of such a system is to support users who have been diagnosed with a specific psychiatric disorder or who suspect they have one and are seeking to develop a coping strategy. For efficient labeling of data to train such a model, it is desirable to automatically pre-classify texts into disorder-specific categories. To achieve this, we utilize data from pre-selected forums and online communities that include source posts describing a user’s situation, attributed by the author to a specific psychiatric diagnosis, along with multiple responses of varying quality and relevance. Text categories (psychiatric disorders) are derived from thread names or source post hashtags and are used as ground truth for evaluating LLM quality and for model fine-tuning.

Zero-shot classification (ZSC) is a machine learning approach that enables a model to recognize new classes without any prior examples. This capability arises because large language models (LLMs) are pre-trained on vast amounts of data, making them relatively accurate in predicting or imitating human responses to queries formulated in natural language. For instance, LLMs can provide reasoned and detailed answers to questions about a text’s topic, specific issues, reasoning methods, and tone, resembling human-generated responses. However, the quality of such classification is often constrained by the lack of relevant texts in the data used to pre-train a given LLM. To address this issue, LLMs are further trained (fine-tuned) on smaller, more relevant datasets, thereby leveraging both extensive general-knowledge data and more focused domain-specific data.

In our work, we explore several approaches to classifying user texts into categories that reflect specific psychiatric disorders. First, we perform pure zero-shot classification (ZSC) into these categories. Second, we precede this ZSC with an additional round of ZSC aimed at filtering out irrelevant texts—those that do not belong to any of the target categories. Third, we fine-tune the LLMs using our dataset. All experiments are conducted on the data retrieved from the Russian language social media.

## Related Work

In this section, we primarily focus on experimental studies and reviews that address the challenges of data availability, quality, and generation in the domain of machine learning for psychiatric purposes. We predominantly reference works from 2023 to 2024, a period marked by significant methodological breakthroughs in LLMs and a corresponding increase in research employing these models for mental health tasks. In this domain, datasets can be categorized into two major types:

- Data about individuals. This includes information on actual or potential mental healthcare recipients and healthy individuals, encompassing their medical records and various digital traces, such as social media posts or data on social networking and app usage. When annotated, these data are labeled with either medically confirmed or self-ascribed diagnoses and are used to train models for classification tasks, such as diagnosis, screening, and risk assessment.
- Data containing mental healthcare seeker queries and responses. The latter can be either human-generated, AI-generated, or both, and are labeled according to criteria such as correctness, professionalism, usefulness, or empathy. These datasets are used to develop dialogue systems.

Most experimental studies, as well as review articles, focus on the efficiency of various algorithms for solving psychiatric tasks, once the data is already available and its quality is considered acceptable. However, several recent review articles highlight data shortages and other data-related issues as significant challenges to the development of LLM applications in psychiatry (Obradovich et al., 2024; Guo et al., 2024; Volkmer, Meyer-Lindenberg & Schwarz, 2024). They include unavailability due to formal privacy regulations protecting relevant data (Obradovich et al., 2024), reluctance of large commercial data owners to share the data (Obradovich et al., 2024), privacy risks for individuals whose data are already in use (Chung, Dyer & Brocki, 2023), lack of non-English datasets and multilingual datasets, especially with expert annotation (Guo et al., 2024), lack of information on or understanding of the datasets when they are available (Guo et al., 2024), and insufficient data quality leading to errors in diagnosis or risk estimation. Other researchers also point at obstacles on the way of creation of high quality datasets. Volkmer, Meyer-Lindenberg & Schwarz (2024) mention uncertainties about creating adversarial examples and difficulties with labeling multi-modal data, which is common in psychiatry. Demszky et al. (2023), although discussing LLM use in psychology, draw attention to a highly relevant problem of low agreement between experts when evaluating the psychological usefulness of AI-generated responses.

According to the available reviews (Hua et al., 2024; Guo et al., 2024), most datasets used in psychiatry-relevant machine learning (ML) are constructed for classification tasks, with text-generation tasks being the second most common. Manual construction is more prevalent than supervised and automatic construction, and datasets annotated by trained laypersons outnumber those annotated by experts. Nearly all datasets are in English, with a few in Chinese and several other languages, ranging from several hundred to a few dozen thousand samples. Social media platforms, especially Reddit and Twitter (now X), are the most common sources of these datasets.

While social media data offer the advantage of availability, they also present certain drawbacks. For instance, they contain little data on older age groups, which might hinder classification tasks (Guo et al., 2024). Similarly, if social media data are used to train psychiatric chatbots, the latter may inherit social prejudices prevalent among internet users whose texts were used for training (Chung, Dyer & Brocki, 2023). Additionally, Aich et al. (2024) observe that the use of large social media datasets often leads to reliance on non-clinical judgments as ground truth in diagnostic tasks, whereas datasets with clinically verified diagnosis labels tend to be very small.

As current proliferation of health-related chatbots is driven primarily by commercial logic, their architectures and the training data often not disclosed. Consequently, existing comparative studies of conversational agents typically do not provide information that would allow attribution of a specific CA’s success to the data it was trained on. Some of these studies are qualitative. For instance, Haque & Rubya (2022) investigated ten commercial mental health chatbots based on over 6,000 consumer reviews analyzed qualitatively. They concluded that the bots generally provided emotionally satisfying communication and immediate help, although they could foster excessive attachment and were not perceived as sufficient replacements for human psychotherapy. Similarly, Martinengo, Lum & Car (2022) found that the nine conversational agents they studied were capable of supportive communication and guiding users in mood-boosting activities. This conclusion was based on the analysis of agent-user interactions by trained assessors using structured criteria.

Other studies employ quantitative approaches. He et al. (2023) conducted a meta-analysis of the efficiency of conversational agent interventions (CAI) based on 32 studies. They demonstrated that CAI improved the symptoms of most investigated psychiatric disorders, including depression, anxiety, and stress, in the short term, but not in the long run. None of these works addressed which CAs were better and why. To the best of our knowledge, Li et al. (2023) is one of the few studies that provides such insights. Based on a meta-analysis of 15 studies involving over 1,500 participants, they found that stronger effects on mental health outcomes were achieved by generative CAs compared to retrieval-based ones, by multi-modal CAs compared to text-only ones, and when delivered *via* mobile devices compared to desktops.

The number of works proposing solutions for the automatic or semi-automatic annotation of mental health data is very limited. As a nascent domain, it is currently developing primarily within non-medical natural language processing (NLP) and for simpler tasks such as named entity recognition (NER), sentiment analysis, and topic and spam detection. Some studies have tested the quality of existing large language models (LLMs) against human annotation, reporting comparable quality for the simplest NLP tasks (Li, 2024; Nasution & Onan, 2024), but lower quality for more complex tasks, such as recognizing six basic emotions (Nasution & Onan, 2024).

The general approach of more advanced methods involves designing human-LLM collaborative systems. These systems typically start by training an annotator LLM on a small amount of human-annotated data. Subsequently, small subsets of LLM-annotated samples are re-annotated by humans and re-submitted as training data for a new round of model training. The final quality of such systems is tested on small held-out datasets that were not involved in any stage of training. This approach was followed by Kholodna et al. (2024) in the NER task; like Nasution & Onan, they experimented with low-resource languages. Wang et al. (2024) took this a step further by introducing an evaluator LLM into the loop, which identified potentially poorly annotated samples to be sent for human re-annotation instead of random samples. Huang et al. (2024) went even further by proposing that LLMs be prompted to develop programs for annotation rather than generating annotations themselves. The goal was to make further annotation cost-free; however, the reported quality varied greatly depending on the task.

The work in the mental health domain most closely related to the task of automated annotation is by Yang et al. (2023). The authors prompted LLMs to determine whether a user was likely to suffer from a given psychiatric disorder based on the user’s text and then to explain the response. By experimenting with zero-shot, few-shot, and emotion-enhanced prompts, they achieved an F1 score between 0.6 and 0.84 on binary classification datasets, but only 0.44 on T-SID multi-class dataset. Further, they compared human and LLM-generated evaluations of the explanations for the obtained labels and found that the overall correlation between human and LLM evaluations was around 0.5. Aich et al. (2024) worked with transcripts of pre-diagnostic interviews conducted by clinicians with patients suffering from bipolar disorder, schizophrenia, and healthy individuals. Interview texts were professionally annotated based on five speech markers indicative of these disorders, such as clarity, focus, and social appropriateness. An LLM was trained to interview individuals and annotate their speech according to these five markers. Fu et al. (2023), while developing a system to help non-professionals conduct online psychological interventions, proposed an idea that could be useful for mental health data annotation. After asking non-professionals to respond to user queries, they prompted an LLM to evaluate the quality of these lay responses. These machine evaluations were further assessed by experienced counselors, who graded 78% to 97% of the LLM-generated evaluations as good, depending on the criterion. This idea of an evaluator LLM is similar to that employed by Wang et al. (2024).

Finally, it is worth noting the absence of relevant works, datasets, or solutions for the Russian language. Although the general-purpose Russian-language LLM, GigaChat (Krestnikov, 2024), has been rapidly developing, no Russian-language mental health CAs exist, except for a Telegram bot proposed by a Kazakhstani team (Omarov et al., 2023). Other CAs developed in countries with Russian-speaking populations are, in fact, English-based. There is one dataset constructed for the early detection of suicidal signals from social media texts (Buyanov & Sochenkov, 2022) and a study predicting well-being and depression risk from multimodal social media and mobile device usage data (Panicheva et al., 2022). Thus, at present, there is no foundation for developing human-LLM annotation systems in the Russian language.

The research gap, thus, can be defined as the lack of cheap fully automated pre-filtering and pre-annotation approaches that could be loosely verified with the existing non-clinical labels and further supplied to professional psychiatrists for expert annotation. This annotation could subsequently be used in mass human-LLM labeling systems leading to mental health CA development. Currently, the described lack is particularly severe for Russian language.

## Zero-shot classification

In this work, we have chosen to apply zero-shot classification (ZSC) because this machine learning technique is well-suited for low-resource tasks. As noted above, in this approach, a model can classify data into multiple classes it has never encountered before, *i.e*., without any specific training examples. This is made possible through the use of auxiliary information; for instance, models can be trained to understand the relationships between different classes and can further transfer this understanding to identify other (unknown) classes based on their similarity (Zhang, Xiang & Gong, 2016). ZSC has been successfully applied in various fields, including natural language processing (NLP) and computer vision (Fu et al., 2018). For example, in NLP, models have been trained to understand semantic relationships between words, which subsequently enabled the models to perform sentiment analysis (Blitzer, Dredze & Pereira, 2007), classify texts by topic (Alcoforado et al., 2022), and execute other tasks without specific training examples. An overview of zero-shot classification models and the tasks where ZSC is used is provided in the articles by Wang et al. (2019), Chen et al. (2021) and Pourpanah et al. (2023).

Based on the works of Fu et al. (2018) and Yin, Hay & Roth (2019), the following modes of ZSC operation can be distinguished.

1) Statement-label mode: This is a straightforward classification mode in which an LLM is tasked to classify statements (or texts) in accordance with pre-determined labels. The essence is that the user can specify the names of the categories by which the data should be classified.
2) Statement-hypothesis mode: This mode can be viewed as a ZSC-adapted application of natural language inference (NLI). NLI is an approach used to test a broad range of hypotheses about text pieces (termed premises), extending beyond simple classification to include assumptions about the logical connections between two phrases, among other things. The NLI task is formulated as binary, where the outcome is either a confirmed or rejected hypothesis. In the NLI adaptation for the classification task, the model tests the hypothesis about whether a text belongs to a given class and determines its probability before yielding a binary response (yes/no). This mode is particularly well-suited for cleaning text collections of irrelevant data.
3) Fine-tuning mode: In this mode, the LLM can be further trained if a relevant dataset is available, enabling it to classify texts more effectively. This can be done in two ways: standard fine-tuning that involves adding a final layer to the LLM and retraining the entire model, and NLI training.

Each of the large language models tested in this work has been run in all three modes.

## Materials and methods

### Models tested

The following two multilingual and two English-only LLMs were used in this study:

- **mDeBERTa-v3-base-xnli-multilingual-nli-2mil7** (https://huggingface.co/MoritzLaurer/mDeBERTa-v3-base-xnli-multilingual-nli-2mil7). This LLM is based on the mDeBERTa-v3-base model pre-trained on the CC100 multilingual dataset, which includes 100 languages.
- **multilingual-MiniLMv2-L6-mnli-xnli** (https://huggingface.co/MoritzLaurer/multilingual-MiniLMv2-L6-mnli-xnli). This multilingual model can perform NLI in over one hundred languages, including Russian, making it suitable for multilingual zero-shot classification. The underlying multilingual-MiniLM-L6 model was created by Microsoft and distilled from XLM-RoBERTa-large. The model was then fine-tuned on the XNLI dataset, which contains hypothesis-premise pairs from 15 languages, as well as the English MNLI dataset (Wang et al., 2020).
- **distilbert-base-uncased-mnli** (https://huggingface.co/distilbert-base-uncased) (Fu et al., 2018). This is an English-language transformer model that is smaller and faster than BERT. It was pre-trained on the same *corpus* in a self-supervised fashion, using the BERT base model as a teacher.
- **DeBERTa-v3-base-mnli-fever-anli** (https://huggingface.co/MoritzLaurer/DeBERTa-v3-base-mnli-fever-anli) (Zhang, Xiang & Gong, 2016). This English-language model was trained on the MultiNLI, Fever-NLI and Adversarial-NLI (ANLI) datasets. The base model is DeBERTa-v3-base from Microsoft (Wang et al., 2020).

These models were chosen based on the following criteria: (1) they are open source which is critical for low-resource languages and societies; (2) they are rated among the most popular zero-shot classifiers by Hugging Face ML platform which hosts them and (3) they have been trained on the largest NLI datasets.

While multilingual models could accept original Russian-language texts, for English-language models the texts were translated using mBART-50 model (https://huggingface.co/docs/transformers/en/model_doc/mbart) (Tang et al., 2020).

### Initial data

The dataset (see Table 1), comprising 64,404 messages published between January 2019 and April 2023, was collected for this research from the following websites:

- **b17 Russian Psychological Forum** (https://www.b17.ru/forum/): (*N* = 48,035);
- **VK Social Network** (https://vk.com, community ‘Psychologists are on the line’, 243K subscribers) (*N* = 15,803);
- **Russian-language Reddit Network** (https://www.reddit.com/): (*N* = 566).

**Table 1:** Message distribution by self-assigned topic class and data sources, unfiltered data.

Topic class	Forum b17	Reddit	VK	Total
Anxiety disorder	8,962		1,107	10,069
Bipolar disorder	2,191	167	604	2,962
Borderline personality disorder	4,585		634	5,219
Depression	28,354	164	9,121	37,639
Neurosis			2,217	2,217
Obsessive-compulsive disorder	3,943	235	1,215	5,393
Paranoia			905	905
Total	48,035	566	15,803	64,404

First, a qualified mental health expert compiled a list of mental disorders. This list was specifically designed to include disorders for which the active adult population is most likely to seek help in an online setting. Consequently, it excluded mental development disorders commonly diagnosed in childhood and aging-related disorders. Substance-related disorders were also left out. The list was roughly based on the F20–F69 categories from the ICD-10 (https://icd.who.int/browse10/2019/en), which is officially adopted in Russia. However, instead of using the exact and nuanced ICD terms, broader and simpler disorder names were included as those that were more likely to be used by non-professional mental health seekers (*e.g*., “paranoia” instead of the respective set of more specific terms).

Second, threads were identified on b17 forum that mentioned one or several of these disorders in their titles. All messages within each of these threads were assigned the label corresponding to the thread title. Third, the categories from the list were used to find messages on Reddit and in the VK *Psychologists are on the line* community using its hashtag search API. In this case, each message had its individual label derived from its hashtag or keyword. Fourth, we selected only the texts from the categories that were sufficiently populated to provide adequate statistics. This selection resulted in a collection of threads with seven single-disorder titles (schizophrenia, anxiety, depression, bipolar disorder, obsessive-compulsive disorder, borderline personality disorder, and paranoia) and one double-disorder title (anxiety/neurosis, further referred to as ‘neurosis’). Schizophrenia texts had to be excluded from the dataset at early stages due to technical issues that prevented effective modeling. As it can be seen from Table 1, class distribution in the remaining dataset is uneven. Choosing among multiple arguments for and against class balancing we prioritize natural class proportions.

The psychological forums and communities were selected because no popular online resources on psychiatry were observed. This is most likely due to the stigmatization of seeking psychiatric support, which is quite prevalent among the general population in Russia, although it is less pronounced in younger cohorts. Self-assigned disorder-related topical class labels were selected as ground truth because a conversational agent aimed at disorder-specific counseling could potentially benefit from being trained on data with a high concentration of discussions about a given disorder. This approach might be effective even if a user’s attribution of their problem to a particular disorder is incorrect. This means that these labels are not considered diagnoses and are not used for diagnostic purposes in this research.

### Data filtration

Preliminary manual text screening revealed that users often did not distinguish between psychiatric disorders and psychological problems when describing their situations. Similarly, messages discussing severe psychiatric symptoms frequently included references to non-psychiatric medical symptoms and conditions. As described below, we test classification architectures both with and without preliminary filtering of obviously irrelevant texts. User text screening has led us to define potentially relevant texts broadly: as those related to any branch of medicine, including psychiatry, or to psychology. As a result of data filtering which is described in more detail in the next section, Neurosis class turned out to have the largest proportion of irrelevant texts and consequently got reduced more than others (see Table 2).

**Table 2:** Message distribution by topic class and dataset type, filtered data.

	Train	Test	Holdout	Total
Anxiety disorder	4,402	943	943	6,288
Bipolar disorder	1,253	269	268	1,790
Borderline personality disorder	2,152	461	461	3,074
Depression	15,229	3,263	3,264	21,756
Neurosis	528	114	113	755
Obsessive-compulsive disorder	2,212	474	474	3,160
Paranoia	361	77	78	516
Total	26,137	5,601	5,601	37,339

### Human labeling

Since the aim if this work is to develop a human-free pipeline for a language where human labeling resources are scarce, humans were not used for labeling either train or test sets, and labels available from the online sources were used as ground truth. However, a limited human annotation was performed for the purposes of overall dataset evaluation and for obtaining insights on using humans for reinforcement learning in the future research. For this, we drew a subsample of texts (*N* = 140, length > 100 characters) from the validation set that included messages from each of seven true classes in equal proportions. Within each true class (except one), texts were evenly distributed between those correctly and incorrectly classified by the best model in its best mode. Two experts with background in psychology were “prompted” similarly to the LLMs. First, they were to identify if the text belonged to medicine, psychology or neither (filtration task), and second, they were to answer seven binary questions on whether each of the disorders was discussed in the given text (classification task). Due to the small size of the dataset and simplicity of the annotation, inter-rater agreement was measured as a share of coinciding responses. It turned to be 94% in the filtration task and 97% to 99% in binary classification questions.

## Computer experiments

The following experiments were conducted on the datasets described above.

### First stage

In the first stage, the quality of the four selected LLMs was evaluated using both the Russian-language and English-language versions of the full dataset. The models were tested as zero-shot classifiers for seven specified categories. Additionally, the impact of the lemmatization procedure on the performance of zero-shot classifiers was assessed by running each of the models on both non-lemmatized and lemmatized texts.

For the lemmatization of Russian-language texts, the **pymorphy** package (Korobov, 2015) was used, while stop-word detection was performed with the NLTK package (Steven, Loper & Klein, 2009). The English-language version of the dataset was lemmatized using the **WordNet** package (Fellbaum, 2005). All lemmatized texts were converted to lowercase, and pseudo-characters without semantic meaning were removed. Documents containing fewer than ten symbols were also excluded. As a result, 63,312 texts were used to test the zero-shot classifiers in the first stage.

### Second stage

In this stage, a zero-shot classifier was applied in the statement-hypothesis mode to filter out irrelevant texts from both the English-language and Russian-language datasets. The classifier tested three binary hypotheses to determine whether each text belonged to one of the following topic classes: psychology, medicine, or other. Each text was assigned to the class with the highest probability, and texts classified as ‘other’ were removed from the dataset.

This classification mode was applied to each of the four models for both the Russian and English versions of the dataset, in their lemmatized and non-lemmatized variations. Since each model performed differently on each dataset version, the resulting datasets varied in size. For instance, the DeBERTa-v3-base-mnli-fever-anli model identified 27,687 medically and psychologically relevant texts out of 63,312 messages, while the mDeBERTa-v3-base-xnli-multilingual-nli-2mil7 multilingual model identified 43,121 texts (see Appendix B for more details in the Supplemental Materials). These filtered datasets were used in the third stage of the calculations.

### Third stage

In the third stage, each model was tested on its respective filtered dataset, which contained only relevant texts. The four models were run in the zero-shot classifier mode.

### Fourth stage

In the final stage, all seven categories were used to fine-tune the LLMs. Since lemmatization did not improve model performance in the earlier stages, we used only non-lemmatized texts that had been identified as relevant by the two models that produced the largest filtered datasets for each language. This resulted in one Russian-language and one English-language dataset, each containing approximately 37,000 texts. For the purpose of fine-tuning, each of these datasets was divided into train, test and holdout parts in the following proportions: 26,137 messages, 5,601 messages and 5,601 messages, respectively. The LLMs were trained and tested on the training and test sets, respectively, and the final evaluation of model performance was conducted on the holdout set. Two types of LLM fine-tuning were applied.

As the first variant we used standard fine-tuning where the output layer with seven neurons was added to the LLM architecture for each of the four models. The entire modified LLM architecture was then trained in a classification mode for seven classes, minimizing the cross-entropy loss function. In this approach, inference was implemented as usual, and the class with the maximum probability was chosen as the final answer.

The second type of fine-tuning involved NLI training, which was adapted for our multiclass task. For this, a binary classification layer (a layer with two output neurons) was added to the model, and the entire model was trained to determine whether each text belonged to any of the seven specified categories, as shown in Table 3. This resulted in one confirmed hypothesis and six rejected hypotheses for each text. After fine-tuning the entire architecture for each of the four models, zero-shot classification using the resulting models was carried out on the holdout set. In all cases during stage four, we used the default prompts recommended for classification tasks in the model codes.

**Table 3:** An example of binary text classifiers for a set of categories.

Hypothesis	Text	Hypothesis is true
Class 1	Text 1	0
Class 2	Text 1	1
Class 3	Text 1	0
…	…	…
Class 1	Text 2	1
Class 2	Text 2	0
…	…	…

## Results

While the experimental results in terms of overall accuracy for all models are summarized in Table 4, Appendices A–D contain more detailed results on performance as measured with multiple metrics (precision, recall, F1 for each class and generalized over all classes as average and as Macro, for each model and dataset).

**Table 4:** Accuracy for all models. For stage 4, holdout dataset results are reported.

Stage	Model parameters	mDeBERTa-v3-base-xnli-multilingual-nli-2mil7	Multilingual-MiniLMv2-L6-mnli-xnli	Distilbert-base-uncased-mnli	DeBERTa-v3-base-mnli-fever-anli
		Russian	Russian	English	English
1	ZSC mode, non-lemmatized texts	0.19	0.12	0.11	0.13
	ZSC mode, lemmatized texts	0.18	0.15	0.11	0.13
2	Filtering, unsupervised	n/a	n/a	n/a	n/a
3	ZSC mode, non-lemmatized texts, filtered	0.22	0.14	0.11	0.19
	ZSC mode, lemmatized texts, filtered	0.20	0.19	0.12	0.19
4	Stanard fine-tuning mode, non-lemmatized texts, filtered	0.63	0.60	0.58	0.61
	NLI fine-tuning mode, non-lemmatized texts, filtered	0.63	0.64	0.57	0.62

### Zero-shot classification on the unfiltered data

The results from applying the four LLMs to the unfiltered data indicate that their classification accuracy is generally low and similar for both lemmatized and non-lemmatized data (see Stage 1 in Table 4). While one multilingual model shows some improvement in classification accuracy on the lemmatized data, the other multilingual model exhibits the opposite trend. Notably, multilingual models tend to perform slightly better in terms of accuracy compared to English-only models. The highest accuracy, approximately 18% to 19%, is achieved by the mDeBERTa-v3-base-xnli-multilingual-nli-2mil7 model. Detailed distributions of all performance metrics by class for each model and dataset are provided in Appendix A in Supplemental Materials.

### Zero-shot classification on the filtered data

As previously described, zero-shot classification into seven disorder-specific topics was applied to datasets from which texts unrelated to the domains of psychology and medicine (including psychiatry) had been filtered out. The results of this further classification on the filtered datasets (see Stage 3 in Table 4) indicate that text filtering using the statement-hypothesis mode generally increases accuracy, albeit modestly, by 1% to 4%. The most significant improvement is observed with the multilingual-MiniLMv2-L6-mnli-xnli model. However, the highest accuracy, as with the unfiltered data, is again achieved by the mDeBERTa-v3-base-xnli-multilingual-nli-2mil7 model, reaching a maximum of 22% on the non-lemmatized data. In most cases, multilingual models outperform monolingual models. Further details on model performance are provided in Appendix B in Supplemental Materials.

### Classification with model fine-tuning on the filtered data

At this final stage, as previously mentioned, both NLI and a conventional approach were applied to fine-tune four language models on two non-lemmatized datasets containing seven topical categories. Table 4 summarizes the accuracy scores obtained on the holdout datasets for these approaches (see Stage 4), while Table 5 includes the results for the training and test sets as well. Both fine-tuning approaches lead to a significant improvement in classification quality. Compared to the results obtained on the filtered data without fine-tuning, the NLI approach increases the classification quality of monolingual and multilingual models by 3.26 times and 3.36 times, respectively. In the case of conventional fine-tuning, the increase in accuracy is 3.15 times for monolingual models and 3.31 times for multilingual models.

**Table 5:** Accuracy of fine-tuned models on the train, test and holdout datasets.

Mode	Dataset	mDeBERTa-v3-base-xnli-multilingual-nli-2mil7	Multilingual-MiniLMv2-L6-mnli-xnli	Distilbert-base-uncased-mnli	DeBERTa-v3-base-mnli-fever-anli
		Russian	Russian	English	English
Standard fine-tuning mode	Train	0.92	0.89	0.93	0.98
	Test	0.63	0.6	0.58	0.61
	Holdout	0.62	0.58	0.57	0.6
NLI fine-tuning mode	Train	0.97	0.89	0.94	0.84
	Test	0.63	0.64	0.57	0.62
	Holdout	0.63	0.64	0.57	0.62

In line with the results of the previous stages, multilingual models outperform English-only models both in terms of absolute accuracy and accuracy gain. Regarding the difference between training modes, NLI fine-tuning yields a slightly larger gain in classification quality compared to the conventional fine-tuning procedure in two out of the four cases. In the other two cases, the gain is either the same or slightly smaller. However, the NLI mode has a significant disadvantage in terms of training time, being six times slower. For example, training a monolingual DeBERTa-v3-base-mnli-fever-anli model takes 2 h in the traditional mode and 12 h in the NLI mode (using the same NVIDIA A100 video card). More detailed results of modeling with conventional and NLI fine-tuning can be found in Appendix C (Supplemental Materials), respectively; full details about LLM computation time are presented in Appendix D (Supplemental Materials).

It should also be noted that we do not observe any significant imbalances between precision and recall that are consistently traceable for any model, classification mode, or topic class. However, the overall quality varies greatly across classes and is closely related to class size. In terms of the F1 score averaged across all fine-tuned models, the following order is observed: depression (0.73), anxiety disorder (0.44), obsessive-compulsive disorder (0.37), borderline personality disorder (0.33), bipolar disorder (0.30), neurosis (0.10), and paranoia (0.06). This distribution provides valuable suggestive evidence about the class size needed for satisfactory classification in the studied domain, indicating that over 20,000 samples, as seen in the depression class, may be necessary.

### Comparison to human evaluation

Although the inter-rater agreement for the filtration task was 94%, experts deemed two-thirds of the cases irrelevant. According to their reports, most texts were responses to user queries that lacked sufficient context to be considered related to either medicine or psychology. Consequently, the agreement between expert opinions and user labels in the classification task was only 29% when calculated on the entire human-labeled subset. This is significantly lower than the best LLM’s performance, which showed 64% agreement on the entire validation test and 78% on the human-labeled subset. While up to one-seventh of the LLM’s advantage over humans could be attributed to chance, the remaining difference suggests that LLMs require less context to predict disorder-specific labels than humans. However, for texts classified as relevant by experts, the agreement between expert opinions and user labels was much higher (78%), and even higher with LLM predictions (90%).

The following conclusions can be drawn from these results. When texts provide sufficient context for human interpretation, LLMs are highly accurate in predicting both expert and user topic labels and can be reliably used for text pre-classification. However, to effectively integrate experts in human-in-the-loop text annotation within the mental health domain, an approach is needed to avoid discarding two-thirds of the texts as irrelevant. Human annotations would benefit from including more context, such as all messages within an N-message left and right context window, as well as the initial user query, if traceable. Given that LLM accuracy also improves with longer texts, this approach could be directly applied in LLM training to enhance their performance.

## Conclusion

In this work, we analyzed the performance of four zero-shot large language models on noisy text data from the mental health domain, with a primary focus on discussions of coping strategies for psychiatric disorders. The goal was to evaluate the ability of LLMs in different modes to pre-filter and pre-classify noisy online data, thereby reducing the workload for annotators in low-resource languages. This task was formulated specifically for the mental health domain and for data that could potentially be used to train disorder-specific mental health conversational agents in the future.

In the following discussion of our results, we compare them to the work of Yang et al. (2023), which is the only known study where LLMs were tested in zero-shot mode to classify psychiatry-related texts. It is important to note that a direct comparison is not possible since the datasets used by Yang et al. (2023) are English-language, noise-free, and mostly two-class. The most relevant three-class dataset, T-SID, contains tweets categorized by high, low, and no suicide risk.

Not unexpectedly and consistent with the findings of Yang et al. (2023), we observe that non-fine-tuned LLMs in ZSC mode perform poorly. The accuracy achieved on our data is 10% lower on the T-SID dataset and does not exceed 0.22. Additionally, we find no improvement in classification quality when using lemmatized data compared to non-lemmatized data, indicating that this preprocessing step can be safely omitted. The superior performance of multilingual models compared to English-only models can be attributed to their ability to directly process Russian-language texts, as they include Russian in their training data. In contrast, translating Russian texts to English may result in information loss. Therefore, translation should only be recommended for languages that lack language-specific LLMs.

Further, we demonstrate that filtering enhances the ability of LLMs to pre-classify data, albeit only by a few percentage points, which falls short of our initial expectations. This limited improvement may be attributed to the fact that the filtering model itself was not fine-tuned, whereas it was fine-tuning that contributed to the quality increase most significantly at the subsequent stage (again, in line with Yang et al. (2023)). The maximum accuracy we achieve with fine-tuned models is 0.64, which is nearly 20% higher than the fine-tuning approach proposed by Yang et al. (2023), although it remains lower than some domain-adapted models explored by Yang et al. (2023). A promising avenue for future research here would be to combine a fine-tuned filtering model with subsequent fuzzy classification, both using domain adaptation. Fuzzy approach could be particularly beneficial, as mental healthcare seekers’ discussions of their coping strategies often encompass multiple existing or alternative diagnoses.

Contrary to our expectations, a dramatic increase in accuracy, approximately 40% to 45%, achieved through model fine-tuning, does not significantly differ between standard fine-tuning and the more advanced Natural Language Inference (NLI) approach. However, the latter results in a substantial increase in computation time, roughly sixfold. Future improvements in NLI fine-tuning could be achieved through more sophisticated hypothesis engineering and incorporating human input, as recently demonstrated for non-psychiatric NLP tasks by Kholodna et al. (2024), Wang et al. (2024), and Huang et al. (2024). It is important to note that straightforward usage of human annotation for reinforcement learning might be not advisable with social media data, and more nuanced approaches to providing context for annotators should be used. Additionally, experimenting with intermediary models to identify potentially poorly annotated samples for further human re-annotation could be beneficial. Existing studies in the mental health domain offer mixed results: such models have shown poor quality in Yang et al. (2023) but high quality in Fu et al. (2023), indicating the need for further experimentation.

In summary, our research makes several significant contributions. We have conducted the first exploration of the potential for LLMs to pre-annotate mental health data in a language for which no relevant annotated datasets currently exist, and for which extensive annotating resources are not anticipated in the near future. Through our investigation of various approaches, we have identified some unnecessary or undesirable steps, such as lemmatization and the use of translated datasets. More importantly, we have pinpointed promising steps, the most notable of which is domain-specific model fine-tuning. Finally, our dataset and fine-tuned models together present a unique resource for the low-resource task of developing a Russian-language conversational agent capable of maintaining disorder-specific mental health counseling dialogues. While our models can be employed as components of such a CA, the dataset can be used for the creation of a vector database for model training and prompt optimization.

## Supplemental Information

10.7717/peerj-cs.2395/supp-1 Supplemental Information 1 Supplementary materials.

10.7717/peerj-cs.2395/supp-2 Supplemental Information 2 Codebook.

## Additional Information and Declarations

### Data Availability

The following information was supplied regarding data availability: The data and python code are available at GitHub and Zenodo: - https://github.com/hse-scila/LLM_psycho_mark_up - ToxaSurkov. (2024). hse-scila/LLM_psycho_mark_up: Code and data for article "Using large language models for psychological data mark-up optimization" (1.1). Zenodo. https://doi.org/10.5281/zenodo.13177432.
