# Supplementary material for: Using large language models for extracting and pre-annotating texts on mental health from noisy data in a low-resource language
Source: PeerJ Comput Sci. 2024 Nov 28;10:e2395. doi: 10.7717/peerj-cs.2395 (PMC11623104; doi:10.7717/peerj-cs.2395)
Supplement: Supplemental Information 1 [file peerj-cs-10-2395-s001.docx]

**Appendix A**

BAD - bipolar affective disorder

BPD - borderline personality disorder

OCD - obsessive-compulsive disorder

Zero-shot classification quality on lemmatized data

|  | Anxiety disorder | BAD | BPD | Depression | Neurosis | OCD | Paranoia | All |
| --- | --- | --- | --- | --- | --- | --- | --- | --- |
| **DeBERTa-v3-base-mnli-fever-anli** |  | | | | | | |  |
| precision | 0.51 | 0.07 | 0.43 | 0.77 | 0.02 | 0.09 | 0.02 |  |
| recall | 0.12 | 0.18 | 0.04 | 0.12 | 0.43 | 0.33 | 0.33 |  |
| f1-score | 0.20 | 0.04 | 0.08 | 0.21 | 0.04 | 0.04 | 0.04 |  |
| Accuracy |  | | | | | | | 0.13 |
| Macro F1 |  |  |  |  |  |  |  | 0.10 |
| **distilbert-base-uncased-mnli** |  | | | | | | |  |
| precision | 0.37 | 0.13 | 0.11 | 0.7 | 0.02 | 0.08 | 0.02 |  |
| recall | 0.10 | 0.03 | 0.03 | 0.08 | 0.32 | 0.45 | 0.08 |  |
| f1-score | 0.16 | 0.05 | 0.05 | 0.14 | 0.03 | 0.14 | 0.03 |  |
| Accuracy |  | | | | | | | 0.11 |
| Macro F1 |  |  |  |  |  |  |  | 0.07 |
| **mDeBERTa-v3-base-xnli-multilingual-nli-2mil7** |  | | | | | | |  |
| precision | 0.09 | 0.09 | 0.79 | 0.06 | 0.11 | 0.03 | 0.32 |  |
| recall | 0.57 | 0.05 | 0.11 | 0.16 | 0.16 | 0.05 | 0.34 |  |
| f1-score | 0.15 | 0.07 | 0.19 | 0.09 | 0.13 | 0.04 | 0.33 |  |
| Accuracy |  | | | | | | | 0.18 |
| Macro F1 |  |  |  |  |  |  |  | 0.14 |
| **multilingual-MiniLMv2-L6-mnli-xnli** |  | | | | | | |  |
| precision | 0.25 | 0.02 | 0.06 | 0.11 | 0.4 | 0.11 | 0.32 |  |
| recall | 0.09 | 0.37 | 0.85 | 0.13 | 0.09 | 0.02 | 0.26 |  |
| f1-score | 0.13 | 0.85 | 0.12 | 0.12 | 0.14 | 0.03 | 0.29 |  |
| Accuracy |  | | | | | | | 0.15 |
| Macro F1 |  |  |  |  |  |  |  | 0.15 |

Zero-shot classification quality on non lemmatized data

|  | Anxiety disorder | BAD | BPD | Depression | Neurosis | OCD | Paranoia | All |
| --- | --- | --- | --- | --- | --- | --- | --- | --- |
| **DeBERTa-v3-base-mnli-fever-anli** |  | | | | | | |  |
| precision | 0.51 | 0.07 | 0.44 | 0.77 | 0.02 | 0.09 | 0.02 |  |
| recall | 0.12 | 0.18 | 0.05 | 0.12 | 0.44 | 0.16 | 0.3 |  |
| f1-score | 0.19 | 0.05 | 0.08 | 0.21 | 0.04 | 0.12 | 0.04 |  |
| Accuracy |  | | | | | | | 0.13 |
| Macro F1 |  |  |  |  |  |  |  | 0.1 |
| **distilbert-base-uncased-mnli** |  | | | | | | |  |
| precision | 0.36 | 0.12 | 0.11 | 0.69 | 0.02 | 0.08 | 0.02 |  |
| recall | 0.11 | 0.03 | 0.03 | 0.08 | 0.31 | 0.46 | 0.08 |  |
| f1-score | 0.16 | 0.04 | 0.05 | 0.14 | 0.03 | 0.14 | 0.03 |  |
| Accuracy |  | | | | | | | 0.11 |
| Macro F1 |  |  |  |  |  |  |  | 0.07 |
| **mDeBERTa-v3-base-xnli-multilingual-nli-2mil7** |  | | | | | | |  |
| precision | 0.32 | 0.13 | 0.1 | 0.76 | 0.03 | 0.12 | 0.02 |  |
| recall | 0.31 | 0.04 | 0.56 | 0.14 | 0.12 | 0.11 | 0.10 |  |
| f1-score | 0.31 | 0.06 | 0.16 | 0.23 | 0.04 | 0.11 | 0.04 |  |
| Accuracy |  | | | | | | | 0.19 |
| Macro F1 |  |  |  |  |  |  |  | 0.12 |
| **multilingual-MiniLMv2-L6-mnli-xnli** |  | | | | | | |  |
| precision | 0.27 | 0.21 | 0.09 | 0.85 | 0.08 | 0.09 | 0.02 |  |
| recall | 0.23 | 0.02 | 0.27 | 0.04 | 0.06 | 0.43 | 0.14 |  |
| f1-score | 0.25 | 0.03 | 0.13 | 0.07 | 0.06 | 0.15 | 0.03 |  |
| Accuracy |  | | | | | | | 0.12 |
| Macro F1 |  |  |  |  |  |  |  | 0.09 |

**Appendix B**

BAD - bipolar affective disorder;

BPD - borderline personality disorder;

OCD - obsessive-compulsive disorder

Zero-shot classification quality on lemmatized filtered data

|  | Anxiety disorder | BAD | BPD | Depression | Neurosis | OCD | Paranoia | All |
| --- | --- | --- | --- | --- | --- | --- | --- | --- |
| **DeBERTa-v3-base-mnli-fever-anli** |  | | | | | | |  |
| Number of filtered obs. | 5082 | 1319 | 2265 | 16458 | 314 | 2385 | 406 | 28229 |
| precision | 0.54 | 0.11 | 0.5 | 0.79 | 0.01 | 0.11 | 0.02 |  |
| recall | 0.19 | 0.15 | 0.06 | 0.21 | 0.54 | 0.12 | 0.29 |  |
| f1-score | 0.29 | 0.13 | 0.11 | 0.33 | 0.03 | 0.11 | 0.05 |  |
| Accuracy |  | | | | | | | 0.19 |
| Macro F1 |  |  |  |  |  |  |  | 0.12 |
| **distilbert-base-uncased-mnli** |  | | | | | | |  |
| Number of filtered obs. | 4105 | 1305 | 1990 | 13228 | 282 | 2059 | 339 | 23308 |
| precision | 0.34 | 0.12 | 0.11 | 0.7 | 0.01 | 0.09 | 0.01 |  |
| recall | 0.14 | 0.05 | 0.06 | 0.08 | 0.3 | 0.45 | 0.04 |  |
| f1-score | 0.2 | 0.07 | 0.08 | 0.15 | 0.03 | 0.14 | 0.02 |  |
| Accuracy |  | | | | | | | 0.12 |
| Macro F1 |  |  |  |  |  |  |  | 0.08 |
| **mDeBERTa-v3-base-xnli-multilingual-nli-2mil7** |  | | | | | | |  |
| Number of filtered obs. | 7217 | 2032 | 3441 | 24586 | 1523 | 3409 | 613 | 42821 |
| precision | 0.33 | 0.11 | 0.09 | 0.8 | 0.08 | 0.11 | 0.04 |  |
| recall | 0.37 | 0.05 | 0.55 | 0.13 | 0.17 | 0.18 | 0.06 |  |
| f1-score | 0.35 | 0.07 | 0.16 | 0.22 | 0.1 | 0.14 | 0.05 |  |
| Accuracy |  | | | | | | | 0.2 |
| Macro F1 |  |  |  |  |  |  |  | 0.15 |
| **multilingual-MiniLMv2-L6-mnli-xnli** |  | | | | | | |  |
| Number of filtered obs. | 1746 | 717 | 1075 | 6291 | 541 | 958 | 189 | 11517 |
| precision | 0.24 | 0.41 | 0.12 | 0.84 | 0.12 | 0.09 | 0.02 |  |
| recall | 0.31 | 0.03 | 0.22 | 0.12 | 0.18 | 0.46 | 0.04 |  |
| f1-score | 0.27 | 0.06 | 0.15 | 0.22 | 0.15 | 0.15 | 0.03 |  |
| Accuracy |  | | | | | | | 0.19 |
| Macro F1 |  |  |  |  |  |  |  | 0.15 |

Zero-shot classification quality on non lemmatized filtered data

|  | Anxiety disorder | BAD | BPD | Depression | Neurosis | OCD | Paranoia | All |
| --- | --- | --- | --- | --- | --- | --- | --- | --- |
| **DeBERTa-v3-base-mnli-fever-anli** |  | | | | | | |  |
| Number of filtered obs. | 3523 | 1816 | 1578 | 18094 | 740 | 1650 | 286 | 27687 |
| precision | 0.44 | 0.15 | 0.45 | 0.82 | 0.03 | 0.08 | 0.02 |  |
| recall | 0.19 | 0.15 | 0.07 | 0.2 | 0.55 | 0.13 | 0.29 |  |
| f1-score | 0.27 | 0.14 | 0.11 | 0.33 | 0.06 | 0.1 | 0.03 |  |
| Accuracy |  | | | | | | | 0.19 |
| Macro F1 |  |  |  |  |  |  |  | 0.12 |
| **distilbert-base-uncased-mnli** |  | | | | | | |  |
| Number of filtered obs. | 8629 | 2602 | 4541 | 32803 | 997 | 4703 | 783 | 55058 |
| precision | 0.37 | 0.12 | 0.12 | 0.7 | 0.02 | 0.08 | 0.02 |  |
| recall | 0.1 | 0.03 | 0.03 | 0.07 | 0.31 | 0.46 | 0.08 |  |
| f1-score | 0.16 | 0.04 | 0.05 | 0.13 | 0.03 | 0.14 | 0.03 |  |
| Accuracy |  | | | | | | | 0.11 |
| Macro F1 |  |  |  |  |  |  |  | 0.07 |
| **mDeBERTa-v3-base-xnli-multilingual-nli-2mil7** |  | | | | | | |  |
| Number of filtered obs. | 7376 | 2038 | 3527 | 25079 | 860 | 3636 | 605 | 43121 |
| precision | 0.33 | 0.16 | 0.1 | 0.77 | 0.04 | 0.12 | 0.03 |  |
| recall | 0.36 | 0.05 | 0.57 | 0.16 | 0.12 | 0.13 | 0.1 |  |
| f1-score | 0.34 | 0.07 | 0.17 | 0.26 | 0.06 | 0.12 | 0.04 |  |
| Accuracy |  | | | | | | | 0.22 |
| Macro F1 |  |  |  |  |  |  |  | 0.13 |
| **multilingual-MiniLMv2-L6-mnli-xnli** |  | | | | | | |  |
| Number of filtered obs. | 1455 | 551 | 854 | 5050 | 252 | 800 | 168 | 9130 |
| precision | 0.24 | 0.17 | 0.1 | 0.8 | 0.09 | 0.09 | 0.03 |  |
| recall | 0.22 | 0.04 | 0.28 | 0.06 | 0.1 | 0.44 | 0.08 |  |
| f1-score | 0.23 | 0.06 | 0.15 | 0.12 | 0.09 | 0.15 | 0.04 |  |
| Accuracy |  | | | | | | | 0.14 |
| Macro F1 |  |  |  |  |  |  |  | 0.11 |

**Appendix C**

BAD - bipolar affective disorder

BPD - borderline personality disorder

OCD - obsessive-compulsive disorder

Classification quality on non lemmatized data with standard fine-tuning

|  | Anxiety disorder | | | BAD | | | | BPD | | | | Depression | | | | Neurosis | | | | OCD | | | | Paranoia | | | | All | |
| --- | --- | --- | --- | --- | --- | --- | --- | --- | --- | --- | --- | --- | --- | --- | --- | --- | --- | --- | --- | --- | --- | --- | --- | --- | --- | --- | --- | --- | --- |
| **DeBERTa-v3-base-mnli-fever-anli** | test | Hold out | | test | | Hold out | | test | | Hold out | | test | | Hold out | | test | | Hold out | | test | | Hold out | | test | | Hold out | | test | Hold out |
| precision | 0.52 | 0.56 | | 0.33 | | 0.34 | | 0.4 | | 0.36 | | 0.68 | | 0.68 | | 0.1 | | 0.09 | | 0.44 | | 0.38 | | 0.16 | | 0.07 | |  | |
| recall | 0.38 | 0.38 | | 0.23 | | 0.24 | | 0.26 | | 0.26 | | 0.83 | | 0.82 | | 0.09 | | 0.08 | | 0.31 | | 0.32 | | 0.12 | | 0.04 | |  |  |
| f1-score | 0.44 | 0.46 | | 0.27 | | 0.28 | | 0.32 | | 0.30 | | 0.75 | | 0.74 | | 0.09 | | 0.09 | | 0.36 | | 0.35 | | 0.14 | | 0.05 | |  |  |
| Accuracy |  | | | | | | | | | | | | | | | | | | | | | | | | | | | 0.61 | 0.6 |
| Macro F1 |  |  |  |  |  |  |  |  |  |  |  |  |  |  |  |  |  |  |  |  |  |  |  |  |  |  |  | 0.34 | 0.32 |
| **distilbert-base-uncased-mnli** |  | | | | | | | | | | | | | | | | | | | | | | | | | | |  | |
| precision | 0.41 | 0.4 | | 0.31 | | 0.27 | | 0.38 | | 0.38 | | 0.69 | | 0.68 | | 0.1 | | 0.11 | | 0.39 | | 0.37 | | 0.17 | | 0.12 | |  | |
| recall | 0.47 | 0.47 | | 0.19 | | 0.18 | | 0.26 | | 0.28 | | 0.76 | | 0.74 | | 0.04 | | 0.05 | | 0.28 | | 0.3 | | 0.03 | | 0.03 | |  |  |
| f1-score | 0.44 | 0.44 | | 0.23 | | 0.22 | | 0.31 | | 0.32 | | 0.73 | | 0.71 | | 0.06 | | 0.07 | | 0.33 | | 0.33 | | 0.04 | | 0.04 | |  |  |
| Accuracy |  | | | | | | | | | | | | | | | | | | | | | | | | | | | 0.58 | 0.57 |
| Macro F1 |  |  |  |  |  |  |  |  |  |  |  |  |  |  |  |  |  |  |  |  |  |  |  |  |  |  |  | 0.31 | 0.3 |
| **mDeBERTa-v3-base-xnli-multilingual-nli-2mil7** |  | | | | | | | | | | | | | | | | | | | | | | | | | | |  | |
| precision | 0.63 | | 0.53 | | 0.39 | | 0.37 | | 0.37 | | 0.36 | | 0.73 | | 0.72 | | 0.17 | | 0.16 | | 0.44 | | 0.4 | | 0.23 | | 0.15 |  | |
| recall | 0.46 | | 0.37 | | 0.36 | | 0.37 | | 0.37 | | 0.36 | | 0.8 | | 0.78 | | 0.15 | | 0.11 | | 0.43 | | 0.42 | | 0.1 | | 0.06 |  |  |
| f1-score | 0.53 | | 0.36 | | 0.37 | | 0.37 | | 0.37 | | 0.36 | | 0.76 | | 0.75 | | 0.16 | | 0.13 | | 0.43 | | 0.41 | | 0.14 | | 0.09 |  |  |
| Accuracy |  | | | | | | | | | | | | | | | | | | | | | | | | | | | 0.63 | 0.62 |
| Macro F1 |  |  |  |  |  |  |  |  |  |  |  |  |  |  |  |  |  |  |  |  |  |  |  |  |  |  |  | 0.4 | 0.38 |
| **multilingual-MiniLMv2-L6-mnli-xnli** |  | | | | | | | | | | | | | | | | | | | | | | | | | | |  | |
| precision | 0.45 | | 0.46 | | 0.42 | | 0.38 | | 0.37 | | 0.32 | | 0.71 | | 0.7 | | 0.17 | | 0.13 | | 0.42 | | 0.4 | | 0.2 | | 0.05 |  | |
| recall | 0.54 | | 0.53 | | 0.25 | | 0.23 | | 0.3 | | 0.3 | | 0.75 | | 0.74 | | 0.16 | | 0.11 | | 0.34 | | 0.32 | | 0.06 | | 0.01 |  |  |
| f1-score | 0.49 | | 0.49 | | 0.31 | | 0.29 | | 0.33 | | 0.31 | | 0.73 | | 0.72 | | 0.16 | | 0.12 | | 0.38 | | 0.36 | | 0.1 | | 0.02 |  |  |
| Accuracy |  | | | | | | | | | | | | | | | | | | | | | | | | | | | 0.6 | 0.58 |
| Macro F1 |  |  |  |  |  |  |  |  |  |  |  |  |  |  |  |  |  |  |  |  |  |  |  |  |  |  |  | 0.36 | 0.33 |

Classification quality on non lemmatized data with NLI fine-tuning

|  | Anxiety disorder | | BAD | | BPD | | Depression | | Neurosis | | OCD | | Paranoia | | All | |
| --- | --- | --- | --- | --- | --- | --- | --- | --- | --- | --- | --- | --- | --- | --- | --- | --- |
| **DeBERTa-v3-base-mnli-fever-anli** | test | Hold out | test | Hold out | test | Hold out | test | Hold out | test | Hold out | test | Hold out | test | Hold out | test | Hold out |
| precision | 0.49 | 0.51 | 0.54 | 0.56 | 0.43 | 0.42 | 0.68 | 0.68 | 0.64 | 0.25 | 0.49 | 0.49 | 0.15 | 0.12 |  | |
| recall | 0.43 | 0.45 | 0.18 | 0.22 | 0.26 | 0.27 | 0.85 | 0.84 | 0.06 | 0.04 | 0.28 | 0.3 | 0.09 | 0.06 |  |  |
| f1-score | 0.45 | 0.48 | 0.27 | 0.32 | 0.33 | 0.33 | 0.76 | 0.75 | 0.11 | 0.06 | 0.36 | 0.38 | 0.11 | 0.09 |  |  |
| Accuracy |  | | | | | | | | | | | | | | 0.62 | 0.62 |
| Macro F1 |  |  |  |  |  |  |  |  |  |  |  |  |  |  | 0.34 | 0.34 |
| **distilbert-base-uncased-mnli** |  | | | | | | | | | | | | | |  | |
| precision | 0.37 | 0.38 | 0.39 | 0.42 | 0.39 | 0.37 | 0.7 | 0.69 | 0.12 | 0.09 | 0.4 | 0.4 | 0.12 | 0.0 |  | |
| recall | 0.47 | 0.49 | 0.2 | 0.21 | 0.28 | 0.28 | 0.74 | 0.73 | 0.09 | 0.07 | 0.32 | 0.32 | 0.04 | 0.0 |  |  |
| f1-score | 0.42 | 0.43 | 0.27 | 0.28 | 0.32 | 0.32 | 0.72 | 0.71 | 0.1 | 0.08 | 0.35 | 0.36 | 0.06 | 0.0 |  |  |
| Accuracy |  | | | | | | | | | | | | | | 0.57 | 0.57 |
| Macro F1 |  |  |  |  |  |  |  |  |  |  |  |  |  |  | 0.32 | 0.31 |
| **mDeBERTa-v3-base-xnli-multilingual-nli-2mil7** |  | | | | | | | | | | | | | |  | |
| precision | 0.56 | 0.55 | 0.44 | 0.56 | 0.46 | 0.47 | 0.71 | 0.71 | 0.15 | 0.2 | 0.46 | 0.44 | 0.24 | 0.22 |  | |
| recall | 0.48 | 0.48 | 0.28 | 0.28 | 0.3 | 0.33 | 0.82 | 0.82 | 0.17 | 0.25 | 0.36 | 0.35 | 0.22 | 0.14 |  |  |
| f1-score | 0.52 | 0.51 | 0.35 | 0.37 | 0.37 | 0.38 | 0.76 | 0.76 | 0.16 | 0.22 | 0.4 | 0.39 | 0.23 | 0.17 |  |  |
| Accuracy |  | | | | | | | | | | | | | | 0.63 | 0.63 |
| Macro F1 |  |  |  |  |  |  |  |  |  |  |  |  |  |  | 0.4 | 0.4 |
| **multilingual-MiniLMv2-L6-mnli-xnli** |  | | | | | | | | | | | | | |  | |
| precision | 0.54 | 0.54 | 0.66 | 0.61 | 0.43 | 0.44 | 0.69 | 0.68 | 0.22 | 0.16 | 0.54 | 0.54 | 0.3 | 0.21 |  | |
| recall | 0.45 | 0.46 | 0.22 | 0.19 | 0.27 | 0.27 | 0.87 | 0.86 | 0.11 | 0.06 | 0.29 | 0.32 | 0.04 | 0.04 |  |  |
| f1-score | 0.49 | 0.5 | 0.33 | 0.29 | 0.33 | 0.34 | 0.77 | 0.76 | 0.14 | 0.09 | 0.04 | 0.41 | 0.07 | 0.07 |  |  |
| Accuracy |  | | | | | | | | | | | | | | 0.64 | 0.64 |
| Macro F1 |  |  |  |  |  |  |  |  |  |  |  |  |  |  | 0.36 | 0.35 |

**Appendix D**

**Resources consumption during fine-tuning procedure**

GPU: nvidia A100

Selected parameters:

1. standard fine-tuning:
   - learning rate (0.000005, 0.00001, 0.00005)
   - resampling (yes/no)
   - linear scheduler with warm up during two epochs (yes/no)
2. NLI fine-tuning: (there's less experimentation, because it takes longer to train)
   - learning rate (0.00001, 0.00005)
   - linear scheduler with warm up during two epochs (yes/no)

Training time per epoch:

|  | DeBERTa-v3-base-mnli-fever-anli (184M parameters) | distilbert-base-uncased-mnli  (67M parameters) | mDeBERTa-v3-base-xnli-multilingual-nli-2mil7 (279M parameters) | multilingual-MiniLMv2-L6-mnli-xnli (107M parameters) |
| --- | --- | --- | --- | --- |
| usual fine tuning | ~2 hours | ~50 minutes | ~ 1 hour 40 minutes | ~ 16 minutes |
| nli fine-tuning | ~12 hours | ~ 4 hours and 40 minutes | ~ 10 hours | ~ 96 minutes |
