# Supplementary material for: Using large language models for extracting and pre-annotating texts on mental health from noisy data in a low-resource language
Source: PeerJ Comput Sci. 2024 Nov 28;10:e2395. doi: 10.7717/peerj-cs.2395 (PMC11623104; doi:10.7717/peerj-cs.2395)
Supplement: Supplemental Information 2 [file peerj-cs-10-2395-s002.docx]

English-language codebook for the non-English text

| депрессия | depression |
| --- | --- |
| тревожное расстройство | anxiety disorder |
| обсессивно-компульсивное расстройство | obsessive-compulsive disorder |
| Пограничное расстройство личности | Borderline personality disorder |
| биполярное аффективное расстройство | bipolar affective disorder |
| невроз | neurosis |
| паранойя | paranoia |
| шаблон гипотезы, например '[LABEL]' или 'этот текст на тему [LABEL]' | a hypothesis template, such as '[LABEL]' or 'this text is about [LABEL]' |
| 'там' | ‘tam’ |
| 'тат' | ‘tat’ |
| добавляет новые строки только к тренировочному датасету | adds new lines only to the training dataset |
| для суперкомпьютера лучше загрузить с гитхаба и указать путь | for a supercomputer, it is better to download from GitHub and specify the path |
